# The Impact of the Wheat *Rht-B1b* Semi-Dwarfing Allele on Photosynthesis and Seed Development Under Field Conditions

**DOI:** 10.3389/fpls.2019.00051

**Published:** 2019-02-04

**Authors:** Emma M. Jobson, Rachel E. Johnston, Alanna J. Oiestad, John M. Martin, Michael J. Giroux

**Affiliations:** Department of Plant Sciences and Plant Pathology, Montana State University, Bozeman, MT, United States

**Keywords:** genetics, grain fill, photosynthesis, plant breeding, wheat

## Abstract

The Reduced Height (*Rht*) genes formed the basis for the green revolution in wheat by decreasing plant height and increasing productive tillers. There are two current widely used *Rht* mutant alleles, *Rht-B1b* and *Rht-D1b*. Both reduce plant height by 20% and increase seed yield by 5–10%. They are also associated with decreased seed size and protein content. Here, we tested the degree to which *Rht-B1b* impacts flag leaf photosynthetic rates and carbon and nitrogen partitioning to the flag leaf and grain during grain fill under field conditions using near isogenic lines (NILs) that were either standard height (*Rht-B1a*) or semi-dwarf (*Rht-B1b*). The results demonstrate that at anthesis, *Rht-B1b* reduces flag leaf photosynthetic rate per unit area by 18% and chlorophyll A content by 23%. *Rht-B1b* significantly reduced grain protein beginning at 14 days post anthesis (DPA) with the greatest difference seen at 21 DPA (12%). *Rht-B1b* also significantly decreased individual seed weight beginning at 21 DPA and by 15.2% at 28 DPA. Global expression analysis using RNA extracted from developing leaves and stems demonstrated that genes associated with carbon and nitrogen metabolism are not substantially altered by *Rht-B1b*. From this study, we conclude that *Rht-B1b* reduces flag leaf photosynthetic rate at flowering while changes in grain composition begin shortly after anthesis.

## Introduction

The introduction of the semi-dwarfing trait into wheat cultivars during the 1960s and 1970s was a defining characteristic of the “Green Revolution” (reviewed in Hedden, 2003). Due to the impressive yield increases associated with these genes, by the late 1990s more than 70% of wheat cultivars grown globally incorporated one of the original semi-dwarfing genes (reviewed in Evans, 1998). The genes associated with the green revolution are mutant forms of the Reduced Height-1 (*Rht*) gene which reduce plant height by decreasing the ability of the plant to respond to GA (Allan et al., 1959; Allan, 1970; Gale and Gregory, 1977). A single functional copy of *Rht* resides on each of the group four chromosomes of wheat (Gale et al., 1975; Gale and Marshall, 1975, 1976; McVittie et al., 1978; Sourdille et al., 1998).

The two most common mutant forms of the *Rht-1* gene are *Rht-B1b* and *Rht-D1b*. Both mutations were introduced into standard height wheat varieties via crosses with semi-dwarf wheat. Both *Rht-B1b* and *Rht-D1b* contain a premature stop codon near the N terminus of the RHT protein (Peng et al., 1999). There is no measurable functional difference between *Rht-B1b* and *Rht-D1b* in that both produce similar increases in wheat productivity and reductions in plant height (Flintham et al., 1997b; Lanning et al., 2012). Since the 1960s many other allelic variants of *Rht-1*, as well as distinctly different genes which also reduce height, have also been discovered. However, none of these mutations have been as useful or as widely incorporated into wheat varieties as *Rht-B1b* or *Rht-D1b*.

The agronomic results seen in varieties carrying either the *Rht-B1b* or the *Rht-D1b* allele is a 15–20% reduction in plant height and increased grain yield (Hoogendoorn et al., 1990; Flintham et al., 1997b). The effects of *Rht-B1b* and *Rht-D1b* are similar in winter and spring wheat (Gent and Kiyomoto, 1997). In all varieties, their advantage is reduced in drought or heat stressed environments, due to decreased seedling emergence (Flintham et al., 1997b) caused by reduced coleoptile length. Due to their decreased height, cultivars carrying *Rht-B1b* or *Rht-D1b* are less prone to lodging, especially under high water and nitrogen conditions (reviewed in Hedden, 2003; Rebetzke et al., 2012; Casebow et al., 2016). Furthermore, different combinations of *Rht-1* alleles can be used to achieve a more diverse range of plant height and agronomic phenotypes. The presence of *Rht-B1b* or *Rht-D1b* alone reduces plant height by 14.6%, but the presence of *Rht-B1b* and *Rht-D1b* together reduces height by 41% (Flintham et al., 1997a). In addition to being shorter than genotypes containing *Rht-B1a* and *Rht-D1a*, lines containing *Rht-B1b* or *Rht-D1b* also have smaller leaves. However, the overall biomass of *Rht-1* semi-dwarf lines is similar to standard height cultivars (Flintham et al., 1997b).

The *Rht-1* semi-dwarfing mutations are also associated with decreased seed size and protein content (Gale and Youssefian, 1985; Appleford et al., 2007; Casebow et al., 2016). However, these studies have only reported differences in grain protein and seed size at maturity, and no studies have investigated when these differences arise during grain fill. Furthermore, very little is known regarding the impact of the semi-dwarfing alleles on seed starch. However, the semi-dwarfing alleles have been associated with decreased alpha amylase activity compared to tall varieties (Van De Velde et al., 2017). Alpha-amylase is a hydrolytic enzyme which can degrade starch. Additionally, it is largely unknown how *Rht-B1b* impacts leaf protein and starch content. One study conducted in the 1970s found that semi-dwarfing lines had increased nitrogen in their stems compared to tall varieties, but that there was no difference in nitrogen translocation efficiency (Deckard et al., 1977).

Despite the widespread use of *Rht-B1b* and *Rht-D1b* in modern wheat varieties, the precise mechanism by which they impact plant growth, seed development, and increase yield is not well understood. One explanation suggests that the yield increase is due to reduced stem elongation and vegetative dry matter accumulation, which leads to increased partitioning of water and nutrients to the spike resulting in increased fertile florets and harvest index (Youssefian et al., 1992) *Rht-B1b* and *Rht-D1b* are also associated with increased productive tillers, which also contribute to increased yield (Kertesz et al., 1991; Lanning et al., 2012; Sherman et al., 2014). Other studies have attributed the increased productivity to increased photosynthetic capacity associated with *Rht-B1b* and *Rht-D1b* (LeCain et al., 1989; Morgan et al., 1990; Bishop and Bugbee, 1998).

Initial studies suggested an inverse relationship between wheat plant height and photosynthetic capacity (LeCain et al., 1989; Morgan et al., 1990; Bishop and Bugbee, 1998). These studies theorized that the decreased cell size in semi-dwarf wheat varieties resulted in a higher concentration of photosynthetic machinery and an increased ratio of chlorophyll containing mesophyll cells compared to non-photosynthesizing cells, which resulted in greater photosynthetic capacity (Morgan et al., 1990). Other earlier studies concluded that in addition to total plant height, flag leaf area was also inversely related to photosynthetic rates (Gale et al., 2009).

However, a more recent study indicates there is no difference in photosynthetic rates between tall and semi-dwarf wheat (Nenova et al., 2014). This study also investigated the impact of the semi-dwarfing genes on seedling leaf structure. Although they reported no difference in net photosynthetic rates between the tall and semi dwarf varieties, the semi-dwarf plants had increased stomatal density and leaf thickness (Nenova et al., 2014). Another recent study compared the photosynthetic capacity of tall plants to the “super dwarf” *Rht-B1c* mutant and again found no difference in photosynthetic rate, but that the dwarf lines had increased chlorophyll content (Dobrikova et al., 2017).

There have been few studies illustrating strong correlations between genetic improvements for yield, and increased photosynthetic rates (Evans, 2013). Due to the prevalence and usefulness of *Rht* dwarfing alleles, it is important to gain a better understanding of how *Rht* alleles increase plant productivity and modify photosynthetic rates. Furthermore, prior experiments investigating the effects of semi-dwarfing alleles on photosynthesis have been conducted in growth chambers which may not be representative of field conditions. It is also important to investigate how the *Rht* semi-dwarf alleles affect assimilate partitioning, and if that also plays a role in increased productivity.

The objectives of these experiments were to investigate the impact of *Rht-B1b* on: photosynthesis of plants grown under field conditions, carbon and nitrogen partitioning in major organs throughout development, and seed development throughout grain fill. From these experiments we hope to observe key differences associated with the *Rht* semi-dwarfing alleles which can later be used to help explain the mechanism by which *Rht* mutations increase plant productivity.

## Materials and Methods

### Plant Material

The NILs used here were previously described (Lanning et al., 2012). The standard height hard red spring variety Fortuna (CI 13596), was used as the recurrent parent line. Fortuna carries the tall, wildtype form of the gene, *Rht-B1a*. The donor semi-dwarf hard red spring parent ‘Hi-Line’ (PI 549275) was used to introduce the *Rht-B1b* allele. Backcrosses to Fortuna continued to the BC_5_ generation, and their genotype was confirmed as described by Ellis et al. (2002). Subsequent generations beyond BC_5_ were allowed to self-pollinate and plants homozygous for the *Rht-B1b* allele were selected for comparison with standard height Fortuna. For this project we excluded the *Rht-D1b* allele; previous studies have shown *Rht-D1b* to be functionally indistinguishable from *Rht-B1b* (Flintham et al., 1997b; Lanning et al., 2012).

### Growing Conditions

Plants were grown under non-limiting irrigated conditions at the Arthur H. Post Field Research Center near Bozeman, MT (latitude 45.67N, longitude 111.00W, elevation 1,455 m, soil type is Amsterdam silt loam). Seeds were planted to a depth of 3.5 cm on April 20th, 2016, and harvested the final week of August. From April 20th to September 1st, the research center received 15.7 cm of precipitation. The highest recorded air temperatures were on July 22 and July 23 at 35.6°C, the lowest recorded air temperature was -2.8°C on May 11, 2016^1^. Additionally, irrigation was applied using hand line sprinklers 1 week pre-and post-anthesis with 5 cm of water applied each time. Throughout the growing season, weeds were rogued out by hand, and the plants were covered by nets to prevent herbivory. The plants were grown in 2.9 m rows with 30 cm spacing between each row. Within a row, seeds were sown 15 cm apart with 19 plants per row. There were 20 rows total, alternating *Rht-B1a* and *Rht-B1b* for a total of 10 rows for each isoline. The entire field was surrounded by four rows of barley to minimize edge effects.

### Plant Sampling

Within each row of 19 plants, five plants were designated and labeled as sampling plants to extract tissue throughout the growing season. Another unique group of five plants were designated and labeled for non-invasive measurements. These plants were randomly selected within each row excluding the plants on the end of each row to minimize edge effects. The five plants selected for tissue collection will be referred to as the “sampling population,” the five plants selected for non-invasive measurements, such as photosynthesis, height, tiller number, and yield, will be referred to as the “non-sampling” population. This was done to ensure that these measurements were not affected by removing or damaging any part of the plant during the growing season. The first five primary heads on each plant to flower from both populations were tagged at heading and used for measurements and tissue collections. This was done to assure that collections and measurements were done on heads of similar maturity. The majority of heads from both lines anthesed on July 6, 2016. If specific heads emerged or anthesed later than the primary heads, it was noted, and collections and measurements were altered to account for this difference.

From the “sampling population” one head and respective flag leaf was collected from each plant at anthesis, as well as at 7, 14, 21, and 28 DPA, and the same plants were used for each collection. The samples from the five plants collectively within one row were considered one biological replicate. All tissue samples were immediately frozen in liquid nitrogen and stored at -80°C. The leaf tissue from these collections was used for: chlorophyll quantification and metabolomic profiling. The grain tissue from these collections was used for: metabolomic profiling and measuring grain protein and starch. For this study, anthesis was defined as the point at which half the primary heads of a plant began extruding anthers. Physiological maturity was denoted as when the primary heads were half brown.

Additionally, one plant from each row was used for starch and protein sampling over a diurnal period. This plant was also randomly selected within each row, excluding the plants on the ends of the row. The first five heads on these plants were also tagged during heading. At 14 DPA, one flag leaf and its respective head from each plant was removed throughout the photoperiod: 30 min before sunrise, 30 min post sunrise, mid-day, and 30 min before sunset (twilight). These time points were chosen to represent the plant’s physiological response after being in total darkness, limited exposure to light, full light, and reduced light. For these experiments, the single plant per row is considered one biological replicate. This plant was not included in any further measurements.

Finally, the plants used for RNA sequencing were grown in unique rows from the rest of the experiment. At 14 DPA leaves and stems were collected from three rows of Fortuna, *Rht-B1b*, and *Rht-D1b*. Within each row, tissue samples were collected from three individual plants and the composite of these three plants from one row was considered one biological replicate.

### Agronomic Measurements

Plant height was measured from the non-sampling population at maturity. It was measured as the distance from the soil surface to the top of the head (excluding the awns) and reported for each plant as the average height of the three tallest tagged tillers. The average from the five plants “non-sampling” plants within the row was considered one biological replicate.

Tiller number at anthesis was measured as the total number of tillers per plant. Measurements were taken on the five “non-sampling” plants within each row, and one biological replicate represents the average of the measurements taken from the five plants per row. Productive tiller number at maturity only counted tillers which set seed. Similarly, each row was considered one biological replicate, and is representative of the average of the measurements taken from five “non-sampling” plants within that row.

Flag leaf length and width were measured on one of the five tagged heads from each of the non-sampling population plants at 14 DPA. Leaf length was measured from the base of the leaf to the tip, leaf width recorded the maximum width of each flag leaf. Each row was considered a biological replicate and represented the average of the measurements from the five non-sampling plants within that row.

Above ground biomass and grain yield were also recorded considering each row as one biological replicate which represented the average of the measurements taken from the non-sampling population within that row. Above ground biomass was measured as the total mass of the plant cut at the soil level and adjusted to a 10% moisture basis. Then, the grain was threshed and weighed and adjusted to a 10% moisture basis for calculations. Harvest index was calculated as grain weight divided by total biomass.

### Photosynthetic Measurements

Photosynthetic measurements were collected at anthesis and 14 DPA from flag leaves on one of the “non-sampling” plants within each row and the measurement from one plant is considered one biological replicate. The measurements were taken at these points in development to illustrate photosynthetic capacity at the beginning and midway through grain fill. The measurements were taken between 13:00 and 15:00 in direct sunlight. The max air temperature at anthesis and 14 DPA was 23.3°C and 30°C, respectively. Data were collected using a CI-340 (CID Bio-Science, Camas, WA, United States) photosynthesis meter according to the methods described in Smidansky et al. (2007) and adapted by Oiestad et al. (2016). This did not include any period of dark adaptation. The leaf area measured was set at 6.5 cm^2^, with a 0.61 min^-1^ flow rate in an open system. Photosynthetic chamber light intensity was held constant using the light attachment module set at 2,000 μE m^-2^ s^-1^ PAR. The light attachment module used red/blue LED lights; the blue peak at 470 nm, and the red peak at 660 nm. Measurements were collected under ambient CO_2_ conditions where the CID photosynthesis meter was calibrated to 404 μL L^-1^ CO_2_ (14 July 2016 and 28 July 2016)^2^. Measurements were recorded every 60 s. Evapotranspiration and stomatal conductance were also calculated using this apparatus (Appendix). Initially, seven measurements were taken on a single leaf to allow the machine to stabilize, then a single measurement was reported per plant to ensure all measurements were taken under similar environmental conditions.

### Chlorophyll Measurements

The tissue for chlorophyll quantification was collected from the “sampling” population within each row at anthesis. One flag leaf from each of the five plants per row was removed and immediately frozen in liquid nitrogen. Later, the five flag leaves from each row were combined and ground into a coarse powder using a mortar and pestle. One biological replicate represents the composite tissue from the five plants within a row. A subsample of the powder was ground into a fine powder using three, three-millimeter diameter glass beads in a two ml tube by beating for 20 s in a bead beater (Biospec Products, Bartlesville, OK, United States). Chlorophyll was quantified using the methods described in Ni et al. (2009) with the quantities adapted for a smaller amount of fresh tissue. To extract the chlorophyll, 0.5 ml of 80% acetone was added to 20 mg of plant tissue powder and agitated. Samples were centrifuged for 5 min and the supernatant was transferred into a new tube, and the process repeated two more times. Total chlorophyll was quantified using a Spectramax spectrophotometer (Molecular Devices, Sunnyvale, CA, United States). The reaction was kept in the dark to prevent the degradation of the chlorophyll. The absorbance was measured at 663 and 646 nm. Chlorophyll was quantified using the following equations from Ni et al. (2009), where *V* is the volume of extract (ml) and *W* is the mass of the leaf tissue (mg).

     Chlorophyll a (μg/g)

          = [12.7 × A663 – 2.69 × A646] × *V*/1000 × *W*

     Chlorophyll b (μg/g)

          =[22.9 × A646 – 4.86 × A663] × *V*/1000 × *W*

     Chlorophyll a and b (μg/g)

          =[20.20 × A646 + 8.02 × A663] × *V*/1000 × *W*

### Grain Weight, Number of Grains per Head, Grain Protein, and Starch Content

Protein and starch content was measured on grain harvested from the “sampling population” within each row at 7, 14, 21, and 28 DPA. At each collection timepoint, one tagged head from each plant within the “sampling population” was harvested and total head fresh weight, individual grain fresh weight, and the number of grains per head was recorded. The samples were then immediately frozen in liquid nitrogen. Later, the grain samples from all five plants within a row were bulked so that one biological replicate represented a composite of all plants within that row. All samples were ground using a mortar and pestle, lyophilized for 24 h in a VirTis BenchTop Freeze Dryer (SP Industries Inc, Warminster, PA, United States) and then allowed to re-equilibrate to ambient moisture (10%). They were then finely ground using a bead beater, as described above. Protein content was quantified using 0.1 g of dry powder on a 10% moisture basis on the LECO – FP 528 (LECO Co., St. Joseph, MI, United States) combustion method nitrogen analyzer using N to protein conversion ratios of 6.25 for seed and 5.7 for leaf tissue (AACC Method 46-30, 1995).

Starch was extracted and quantified as described by Smith and Zeeman (2006) and adapted by Schlosser et al. (2014). One ml of 80% ethanol was added to 10 mg each plant sample in a 2-ml tube and the samples were incubated at 80°C for 3 min while mixing at 1,400 rpm. After 3 min, the samples were centrifuged at 13,000 × *g* for 5 min and the supernatant was discarded. This process was repeated two additional times and the samples were dried for 1 h in a speed-vac concentrator (Fisher Scientific, Waltham, MA, United States). The pellets were re-suspended in 100 mM sodium acetate (pH 4.8). The starch was digested as described by Schlosser et al. (2014) using 0.05 U α-amylase and 0.15 U amyloglucosidase mg^-1^ dry weight. Starch was quantified as described by Rosti et al. (2006) using a standard curve based on known amounts of purified wheat seed starch.

### Flag Leaf Protein and Starch Content

Flag leaf protein and starch were quantified using the same methods as described above at 14 DPA. However, the flag leaf tissue was collected over a diurnal cycle (30 min pre-sunrise, 30 min post-sunrise, mid-day, and 30 min pre-sunset), rather than 7, 14, 21, 28 days DPA. Furthermore, measurements for flag leaf protein and starch were all collected from a single plant per row, rather than the five plants of the “sampling population.” The first five heads of the plant to be sampled were tagged during heading, and 14 DPA each head and respective flag leaf were removed throughout the diurnal cycle. Once collected, the tissue was immediately frozen, lyophilized, and ground as described above.

### Expression Analysis

Tissue was collected for expression analysis from stems between the first and second internode as well as 14 DPA flag leaves at mid-morning. A single leaf and internode was sampled from three distinct *Rht-B1a* and *Rht-B1b* plants from different rows, for a total of three biological replicates for each line per tissue type. Total RNA was extracted and sequenced as described in Oiestad et al. (2016) using an RNeasy Plant Mini Kit (Qiagen, Valencia, CA, United States) and quantified with a Bioanalyzer (Agilent Technologies, Santa Clara, CA, United States). One mg of RNA was used to create a cDNA library using TruSeq RNA-SEQ library kits (Illumina Inc., San Diego, CA, United States). The sequence data were analyzed using ArrayStar (DNASTAR, Madison, WI, United States), with the parameters set as: match setting at 100% for a minimum 50 bp, and all other settings left as default. The resultant data were presented as reads per kilobase of transcript for million mapped reads (RPKM) (Mortazavi et al., 2008) and normalized to *Act-2*, an actin like protein which was similarly expressed in both genotypes, and previously reported as a reliably expressed gene for normalization (Tenea et al., 2011). The data was analyzed globally against the available rice genome. Specific wheat genes of interest were also analyzed. Genes central to either carbon or nitrogen metabolism were identified based on expression profiling done in rice by Hirose et al. (2006) and adapted in Schlosser et al. (2014) to identify the most prevalent form of the gene in green tissue during development. Wheat genome orthologs were identified using NCBI BLAST^3^ and used for expression analysis.

### Leaf and Grain Metabolites

Tissue for metabolite analysis was collected from the flag leaves at anthesis and 14 DPA seeds from the “sampling population.” The leaf and grain tissue from each individual row was bulked together to create four or five biological replicates for each genotype for each tissue. Metabolites were extracted as described by Schmidt et al. (2011) and adapted by Oiestad et al. (2016). The tissue was frozen in liquid nitrogen and ground to a fine powder as described above, then 350 μl of methanol (75°C) was added to 20 mg of each sample and incubated at 60°C for 10 min. After the samples were vortexed, they were incubated in a sonicating water bath for 10 min. After 10 min, 350 μl of chloroform was added to each sample and vortexed. Finally, 300 μl of ddH_2_O was added and the samples were vortexed, then centrifuged at 13,000 × *g* for 5 min. The polar fraction was transferred to a GC-MS glass vial in a volume dependent manner (150 mL per 30 mg FW) and dried in a speed-vac concentrator. Samples were analyzed on an Agilent 6890 gas chromatograph (Agilent Technologies). Data acquisition, metabolite identification and normalization were performed as in Fiehn et al. (2008).

### Statistical Analysis

Statistical analysis to compare the difference between the *Rht-B1a* and *Rht-B1b* allelic groups was done using a two-tailed, paired sample *t*-test. A paired *t*-test was used because the two genotypes were paired in adjacent rows. The number of replications (rows) and subsamples varied for each experiment.

## Results

### Agronomic Measurements

As expected, there was a significant height reduction (*P* < 0.001) between *Rht-B1b* and *Rht-B1a* under the field conditions used. The *Rht-B1b* NIL had a 23% reduction in height (Table 1). No difference was observed in productive tiller numbers between *Rht-B1b* and the *Rht-B1a* in this study in which plants were space planted 15 cm apart. Plants carrying *Rht-B1b* had a decreased biomass (by 16.4%) and increased harvest index (17.2%). We also observed a significant (*P* < 0.0001) decrease in flag leaf length in the *Rht-B1b* line compared to *Rht-B1a*, 12.6 cm and 14.7 cm, respectively. There was also a significant (*P* < 0.05) decrease (12%) in flag leaf width.

**Table 1:** Comparison of plant height, flag leaf dimensions, tiller number, and biomass between *Rht-B1a* (wild-type) and *Rht-B1b* (mutant) near isogenic lines.

	*N*	*Rht-B1a* (wild-type)	*Rht-B1b* (mutant)
Plant height (cm)	13	95.0 ± 1.35	73.1 ± 0.19***
Tiller no. at anthesis	13	24.0 ± 1.98	23.7 ± 1.71
PTN^‡^ at maturity	13	20.0 ± 0.60	19.1 ± 0.60
Flag leaf length (cm)	10	14.7 ± 0.37	12.6 ± 0.31***
Flag leaf width (mm)	10	13.9 ± 0.28	12.5 ± 0.21*
Biomass (g/plant)	10	53.0 ± 2.24	44.3 ± 1.83***
Harvest index	10	0.29 ± 0.01	0.34 ± 0.01***
Grain yield (g/plant)	10	15.4 ± 0.74	15.4 ± 0.68

^∗^, ^∗∗∗^*denote significance between genotypes (*t*-test) at *P*-value < 0.05, 0.001, respectively. Values reported as the mean ± standard error. ^‡^PTN, productive tiller number. For height and tiller measurements, *n* represents the average of three plants measured per row; for the remaining measurements, *n* represents the number of rows where five plants were measured and averaged from an individual row; plant height measurements were measured at physiological maturity*.

### Photosynthetic Capacity

**Figure 1:**
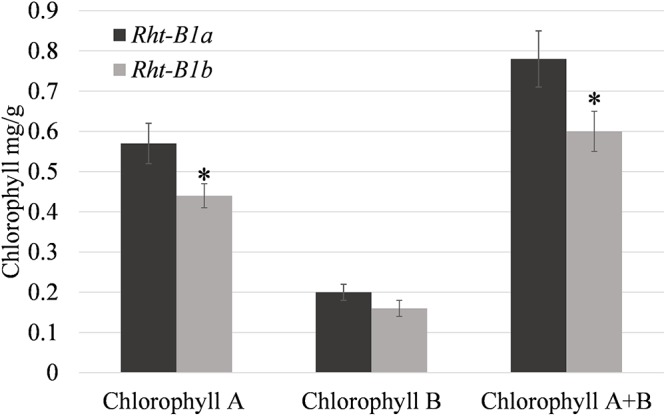
Relative amounts of chlorophyll in flag leaf tissue at anthesis for *Rht-B1a* (wild-type) and *Rht-B1b* (mutant) near isogenic lines. ^∗^ denotes significance at *P*-value < 0.05 in comparisons of *Rht-B1a* vs. *Rht-B1b*, *N* = 4, where *n* represents the composite of five sampling plants per row, error bars represent the standard error.

Photosynthetic rates were measured in the field at anthesis and at 14 DPA. At both time points, photosynthetic rates trended down in the *Rht-B1b* NIL (Table 2), and were significantly decreased (17.8%, *P* < 0.01) at anthesis. Evapotranspiration and stomatal conductance were also decreased to a similar degree (13.7 and 20.2%, respectively, *P* < 0.05) at anthesis and trended lower at 14 DPA in the *Rht-B1b* NIL. Similarly, *Rht-B1b* flag leaf chlorophyll trended lower relative to *Rht-B1a* (Figure 1). The difference between *Rht-B1b* and *Rht-B1a* was pronounced in chlorophyll A, the primary molecule responsible for photosynthesis, (23% reduction in *Rht-B1b*).

**Table 2:** Photosynthetic rates of *Rht-B1b* (mutant) and *Rht-B1a* (wild-type) NILs measured as carbon exchange in flag leaves at two stages of development.

	*N*	Photosynthetic rate (μmol m^-2^s^-1^)	Evapotranspiration (mmol m^-2^s^-1^)	Stomatal conductance (mmol m^-2^s^-1^)
**Anthesis**				
*Rht-B1a* (wild-type)	10	23.7 ± 0.84	5.12 ± 0.22	160.0 ± 9.56
*Rht-B1b* (mutant)	10	19.5 ± 1.09**	4.42 ± 0.11*	127.6 ± 9.41*
**14 DPA**				
*Rht-B1a* (wild-type)	6–9	10.3 ± 0.79	4.08 ± 0.24	63.0 ± 5.98
*Rht-B1b* (mutant)	5–8	8.3 ± 1.38	3.18 ± 0.43	57.0 ± 2.88

^∗^, ^∗∗^*denote significance between genotypes at *P*-value < 0.05 and 0.01, respectively. Values reported as the mean ± standard error. *N* represents the measurements taken from one flag leaf per row; all measurements were collected on flag leaves in direct sunlight*.

### Leaf Protein and Starch Content Throughout Photoperiod

**Figure 2:**
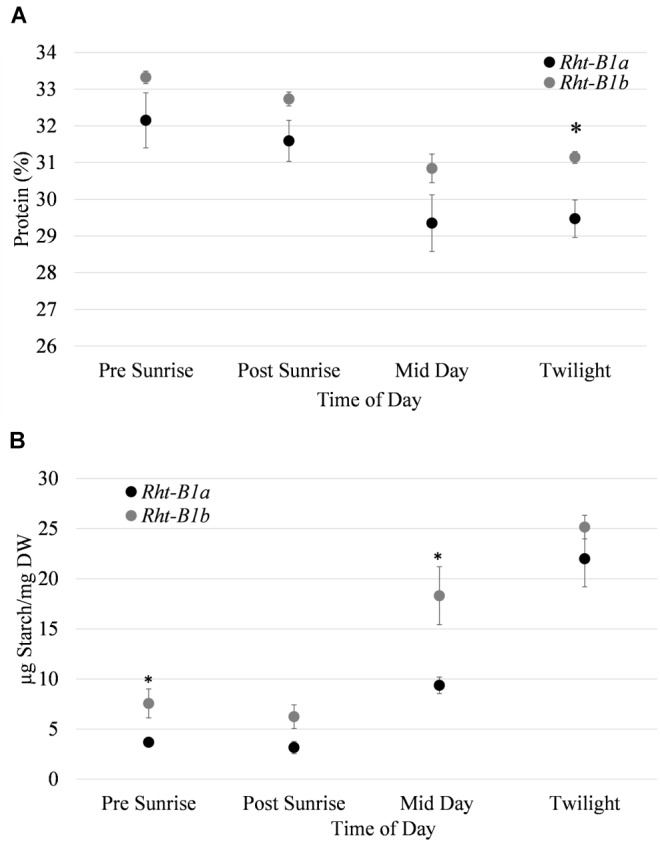
Abundance of protein and starch quantified in flag leaves over a diurnal period at anthesis for *Rht-B1a* (wild-type) and *Rht-B1b* (mutant) near isogenic lines. **(A)** Leaf protein abundance throughout photoperiod. **(B)** Leaf starch throughout diurnal period. ^∗^ denotes significance at *P* < 0.05 in comparisons of *Rht-B1a* vs. *Rht-B1b*, for **(A)** *N* = 4, where *n* represents the composite of five flag leaf samples taken per row; error bars represent the standard error. For **(B)** *N* = 5, where *n* represents the composite of five flag leaf samples taken per row error bars represent the standard error.

Flag leaf protein and starch content were recorded throughout the photoperiod during anthesis (Figure 2) at 30 min prior to and after sunrise, mid-day (14:00) and 30 min pre-sunset. For all time points, leaf protein content trended upward in the *Rht-B1b* NIL, with a significant difference (1.67%, *P* < 0.05) observed at twilight. While total protein as predicted by N content was different, there was no difference in the abundance of the major photosynthetic proteins as quantified by SDS PAGE (results not shown) between *Rht-B1a* and *Rht-B1b*.

Flag leaf starch content was also elevated in *Rht-B1b* compared to *Rht-B1a*. The greatest difference in leaf starch content between the two NILs was observed in flag leaves collected at mid-day. At that point, there was 18.3 μg starch/mg dry weight in *Rht-B1b* (mutant) and 9.4 μg starch/mg dry weight in the *Rht-B1a* (*P* < 0.05) (Figure 2B). The *Rht-B1b* NIL had the greatest increase in starch between the post sunrise and mid-day measurements increasing from 6.24 to 18.31 μg starch/mg dry weight, and the *Rht-B1a* variety had its greatest increase of starch production between the mid-day to twilight measurements, increasing from 9.37 to 22 μg starch/mg dry weight.

### Starch and Protein Content of Grain Throughout Development

The impact of *Rht-B1b* upon seed starch and protein content during development was measured to assess when differences first develop. Developing seeds were isolated by harvesting heads from primary tillers at 7, 14, 21, and 28 DPA. Starch and protein content in developing seeds through maturity at 28 DPA is summarized in Table 3. There were no significant differences in seed starch content between the *Rht-B1a* and *Rht-B1b* NILs throughout development. However, protein content was reduced in *Rht-B1b* relative to *Rht-B1a* NILs throughout development. The difference in seed protein content was statistically significant beginning at 14 DPA when protein content was measured as 13.1% in *Rht-B1a* and 12.0% in *Rht-B1b (P* < 0.01). This observed difference between the isolines was consistent through maturity. At 28 DPA the *Rht-B1b* line had a 1.83% decrease in protein content compared to *Rht-B1a* (*P* < 0.05).

**Table 3:** Starch and protein content in grain throughout development for *Rht-B1a* (wild-type) and *Rht-B1b* (mutant) near isogenic lines.

	*N*	7 DPA	14 DPA	21 DPA	28 DPA
Percentage starch					
*Rht-B1a* (wt)	5	36.3 ± 1.93	50.1 ± 0.74	68.6 ± 3.73	66.6 ± 3.00
*Rht-B1b* (mt)	5	32.9 ± 0.97	50.6 ± 3.95	64.8 ± 1.99	66.3 ± 1.58
Percentage protein					
*Rht-B1a* (wt)	5	10.0 ± 0.32	13.1 ± 0.25	12.4 ± 0.29	13.3 ± 0.23
*Rht-B1b* (mt)	5	10.1 0.33	12.1 ± 0.10**	11.0 ± 0.16**	12.0 ± 0.33*

^∗^, ^∗∗^*denote significance between genotypes at *P*-value < 0.05, 0.01, respectively. Values reported as the mean ± standard error. *N* = 5, where *n* represents the number of rows where each row was a composite of five sampling plants*.

### Seed Number and Individual Kernel Weight Throughout Development

**Figure 3:**
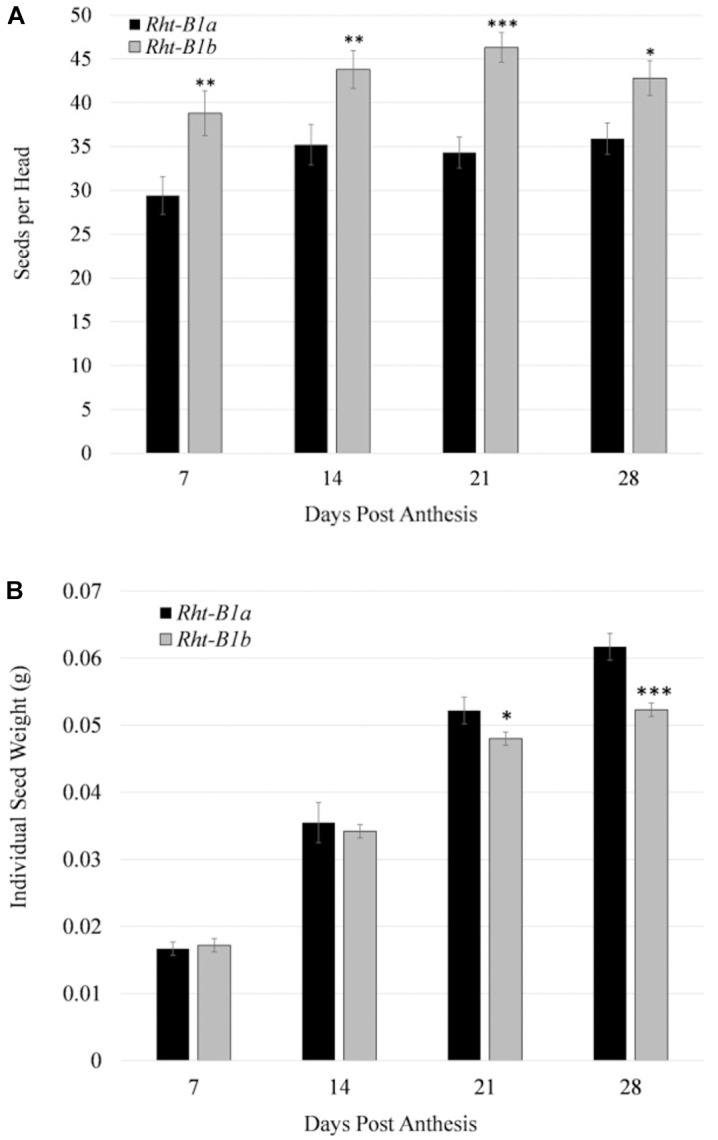
Comparison of grain number and size throughout grain fill for *Rht-B1a* (wild-type) and *Rht-B1b* (mutant) near isogenic lines. **(A)** Number of seeds per head throughout grain fill. **(B)** Individual seed mass over grain fill. ^∗^, ^∗∗^, ^∗∗∗^ denote *P*-value at 0.05, 0.01, 0.001, respectively; *N* = 10, where *n* represents tissue collected from one plant per row; error bars represent the standard error.

Three aspects of grain fill were measured, these being the average seed per head, total weight of the head, and individual seed fresh weight (Figure 3). Beginning at 7 DPA, the *Rht-B1b* NIL had an increased seed number per head compared to the *Rht-B1a*, (38.8 versus 29.4). Initially, total head weight was also increased in the *Rht-B1b* NIL compared to *Rht-B1a*. However, by 28 DPA both NILs had an average head weight of 2.2 g. The individual kernel weight was consistently greater in *Rht-B1a* beginning at 14 DPA. The final individual grain fresh weight for *Rht-B1a* and *Rht-B1b* NILs were 61.7 mg and 52.3 mg/seed, respectively.

### Effect of *Rht-B1b* Mutation on Metabolite Production

Total methanol soluble metabolites were extracted from flag leaf tissue harvested at anthesis and seeds at 14 DPA at 10:00. One hundred and twenty-seven compounds were identified in the leaf, and 126 compounds were identified in the grain. The metabolites which were identified were grouped into four different categories: fatty acid, sugar and sugar alcohols, amino acid derivatives, and amino acids. There were no major differences observed among these four groups between *Rht-B1b* and *Rht-B1a*, suggesting *Rht-B1b* does not have a widespread global effect on plant metabolism.

### Expression Analysis of Leaf and Stem RNA

RNA sequencing data was analyzed globally as well as for the expression of genes involved in photosynthesis, carbon metabolism, and nitrogen metabolism (Table 4). The presence of *Rht-B1b* did not cause significant global gene expression changes in leaf tissues. However, it did impact gene expression in stem tissue. In regard to photosynthesis, the RuBisCO small subunit was significantly increased in *Rht-B1b* stem tissue (*P* < 0.01). Genes associated with carbon metabolism in stems were also generally upregulated in *Rht-B1b*, including starch synthase (*P* < 0.04). Genes involved in nitrogen metabolism were less effected, though glyeraldehyde-3-phosphate dehydrogenase was increased significantly in *Rht-B1b* (*P* < 0.04). The data discussed in this publication have been deposited in NCBI’s Gene Expression Omnibus (Edgar et al., 2002) and are accessible through GEO Series accession number GSE124940^4^.

**Table 4:** Gene expression values in leaves and stems of genes involved in photosynthesis and assimilate partitioning.

		Stems				Leaves			
Name	GenBank accession number	*Rht-B1a* average RPKM	*Rht-B1b* average RPKM	*P*-value	*Rht-B1b/ Rht-B1a*	*Rht-B1a* average RPKM	*Rht-B1b* average RPKM	*P*-value	*Rht-B1b/ Rht-B1a*
Carbon metabolism									
Granule-bound starch synthase (*GBSSII*)	AF109395	1146 ± 109	1978 ± 283	0.09	1.7	1457 ± 259	1465.5 ± 190.5	0.98	1.0
Starch branching enzyme (*Sbe2*)	AF286319	230 ± 17	345 ± 41	0.10	1.5	329.0 ± 66.6	318.7 ± 33.7	0.92	0.9
Sucrose transporter (*SUT1D*)	AF408845	974 ± 131	887 ± 50	0.64	0.9	13194.8 ± 1224.2	16627.5 ± 870.6	0.14	1.3
ADP-glucose pyrophosphorylase large subunit (*agp2*)	AJ563452	1657 ± 202	3785 ± 613	0.05	2.3	365.6 ± 37.8	304.0 ± 26.8	0.34	0.8
starch synthase isoform IV	AY044844	378 ± 27	589 ± 50	0.04	1.6	880.2 ± 94.3	1065.9 ± 104.1	0.34	1.2
ADP-glucose pyrophosphorylase small subunit	AY727927	1236 ± 197	1599 ± 86	0.24	1.3	1535.9 ± 402.1	897.3 ± 185.0	0.30	0.6
Starch branching enzyme (*sbeiia*)	HE591389	2112 ± 259	2405.9 ± 269.2	0.56	1.1	2508.7 ± 492.2	2798.4 ± 315.4	0.71	1.1
Sucrose transporter (*SUT2D*)	KJ812205	559 ± 27	579.6 ± 12.8	0.60	1.0	725.1 ± 54.6	686.3 ± 46.2	0.68	0.9
RuBisCO small subunit (*rbcS*)	AB042066	85754 ± 1267	125966.0 ± 5180.2	0.00	1.5	1166680.2 ± 289990.1	1250289.4 ± 150391.2	0.84	1.1
**Nitrogen metabolism**									
Ferredoxin dependent glutamate synthase (*NADH-GOGAT-3B*)	KC960544	2818.5 ± 83.0	2083.5 ± 380.9	0.20	0.7	434.9 ± 17.0	424.8 ± 25.2	0.80	0.9
Glyeraldehyde-3-phosphate dehydrogenase (*GAPC8*)	KR029493	65648.5 ± 8387.6	101553.1 ± 4195.1	0.04	1.6	39053.0 ± 2791.0	40016.2 ± 3241.7	0.86	1.0
**Rht genes**									
*Rht-D1*	AJ242531	1311.9 ± 60.7	1739.5 ± 123.0	0.06	1.3	707.8 ± 81.2	808.1 ± 18.4	0.38	1.1
*Rht-B1*	FR668586	1823.0 ± 40.7	4512.2 ± 165.2	0.00	2.5	1116.2 ± 146.4	2407.4 ± 247.5	0.02	2.2
**Housekeeping gene**									
Actin	AB181991	1432.5 ± 0.0	1432.5 ± 0.0			1432.5 ± 0.0	1432.5 ± 0.0		

*Data represents the average ± the standard error and is reported as the reads per kilobase million (RPKM), *n* = 3, *P*-value represents a two-tailed paired *t*-test, expression normalized to actin (Tenea et al., 2011)*.

## Discussion

Incorporation of height reducing (*Rht*) semi-dwarf genes *Rht-B1b* and *Rht-D1b* into wheat cultivars has led to dramatic increases in grain yield (Flintham et al., 1997b). Mutant forms of the Reduced Height-1 (*Rht*) gene reduce plant height by decreasing the ability of the plant to respond to GA (Allan et al., 1959; Allan, 1970; Gale and Gregory, 1977) However, the underlying causal factor for the increased productivity, and the effects on plant growth and development is has not yet been fully characterized. To understand the effects of the semi-dwarfing alleles on plant growth and development more completely, we investigated the effect of *Rht-B1b* on photosynthesis as well as carbon and nitrogen partitioning during grain fill under field conditions.

Our results show the *Rht-B1b* NIL had reduced plant height, increased harvest index and decreased grain protein compared to *Rht-B1a*, NIL (Table 1). These results agree with Lanning et al. (2012) who used the same Fortuna derived NILs as this study. These results are consistent with previous studies which reported 20–25% height reductions (Hoogendoorn et al., 1990; Butler et al., 2005; Mathews et al., 2006; Lanning et al., 2012). Previous experiments have also described an increase in productive tillers, leading to increased yields in *Rht-B1b* genotypes (Kertesz et al., 1991; Lanning et al., 2012; Sherman et al., 2014). Lanning et al. (2012) reported a 9% grain yield advantage for *Rht-B1b* (mutant) over the *Rht-B1a* for these NILs from solid seeded conditions. We did not detect a difference in grain yield. This may have been because our trial was grown in spaced planted conditions, which would have reduced resource competition for both NILs.

The effects of *Reduced Height* genes on photosynthesis have been inconclusive. Morgan et al. (1990) reported increased photosynthetic rates, increased soluble protein, chlorophyll, and RuBisCO content in semi-dwarf wheat when compared to near isogenic tall lines. It has also been previously thought that there was an inverse relationship with photosynthesis and wheat plant height (LeCain et al., 1989; Bishop and Bugbee, 1998). However, when Nenova et al. (2014) compared photosynthetic rates of NILs, they found no significant difference between the semi-dwarf and tall lines. None of these studies investigated the photosynthetic capacity of the *Rht-B1b* lines compared to tall *Rht-B1a* under field conditions and using NILs. Our results indicate that photosynthesis per unit area is decreased in the *Rht-B1b* line compared to *Rht-1a* (Table 2). We observed a decrease in both photosynthetic rate and chlorophyll (Table 2 and Figure 1) content in the *Rht-B1b* NIL. However, these results do not take into account the total photosynthetic capacity of the plant canopy, and our experiment did not measure the total leaf area per plant. Additionally, our experiment did not observe increased tiller number, which have been previously associated with *Rht-B1b*. Plants with relatively more tillers would likely have a greater net photosynthetic capacity compared to plants with fewer tillers. From the experiments we conducted, we can conclude that when grown under space planted, irrigated field conditions, *Rht-B1b* decreases photosynthetic rate per unit area compared to the tall *Rht-B1a* during early grain development.

Although the photosynthetic rate and chlorophyll content of *Rht-B1b* was decreased, we observed an increase in both leaf starch and protein concentration for both genotypes during the grain fill period. Previous studies have documented carbohydrate accumulation in leaves to decrease the expression of photosynthetic genes, and therefore photosynthesis (Paul and Foyer, 2001). It is possible that since the *Rht-B1b* line had increased leaf starch content, this was inhibiting the photosynthetic capacity. Furthermore, research has suggested that inactivation of sucrose symporters may also reduce photosynthesis, and would increase the amount of starch in the leaf (reviewed in Sukhov, 2016).

While the grain starch and protein content have been previously characterized at maturity, the leaf starch and protein content throughout a diurnal period during anthesis was unknown. It appears that presence of the *Rht-B1b* allele resulted in starch accumulating in flag leaves earlier in the day relative to *Rht-B1a* (Figure 2). However, by the end of the day, there was no significant difference between *Rht-B1b* and *Rht-B1a*. We observed no significant difference in protein content of flag leaves throughout the diurnal period.

*Rht-B1b* has been previously associated with decreased grain protein content, (Gale and Youssefian, 1985; Lanning et al., 2012; Sherman et al., 2014). However, it is unknown at what point during grain development the differences in protein arise. Lanning et al. (2012) showed *Rht-B1b* had mature seed protein 1.1% lower than the *Rht-B1a*. Our results agree with this finding and show that there is a significant reduction in grain protein content beginning 14 DPA and continuing through maturity (Table 3). We did not observe significant differences in seed starch content between *Rht-B1b* and *Rht-B1a*. It has also been previously determined that varieties containing *Rht-B1b* have a greater number of seeds per spike, but that the seeds are smaller (reviewed in Gale and Youssefian, 1985), and have decreased protein content at maturity (Lanning et al., 2012). Our results support these findings as well as indicate that those differences are present beginning at 14 DPA, and that the differences increase as plants mature (Figure 3).

We also investigated the effect of *Rht-B1b* on leaf and seed metabolites and global gene expression, which had both been previously uncharacterized. We detected no significant changes in leaf or seed metabolites, indicating there was likely no significant difference in carbohydrates due to differences in photosynthesis or carbon metabolism. Furthermore, RNA sequencing analysis showed no difference in leaf photosynthetic gene expression between *Rht-B1b* and *Rht-B1a* (Table 4). However, there were significant differences in expression detected in stem tissue. Genes involved in photosynthesis and carbon metabolism were upregulated in the *Rht-B1b* stem tissue. Specifically, *agp2*, a gene encoding the ADP-glucose pyrophosphorylase large subunit was upregulated in *Rht-B1b*. Upregulation of *agp2* has been associated with increased starch content (Zhang et al., 2016). This may partially explain the increased starch content in the *Rht-B1b* lines. We also found the RuBisCO small subunit was significantly increased in the stem tissue of the *Rht-B1b* line. Global expression analysis did not indicate any large groups of differentially expressed genes (data not included), indicating *Rht-B1b* likely has little effect on global gene regulation.

It is clear that the presence of the *Rht-B1b* semi-dwarfing alleles has dramatic effects on wheat plant growth and development, and that those effects begin during early development and continue throughout development. At anthesis *Rht-B1b* NIL plants had decreased photosynthesis and chlorophyll content in flag leaves. *Rht-B1b* NIL plants also had reduced grain protein content and size as early as 14 DPA. However, despite these changes in plant growth and development, we did not detect any significant changes in global gene expression due to the presence of the semi-dwarfing allele.

## Author Contributions

EJ, JM, and MG conceived and designed the experiments and wrote the paper. EJ, RJ, and AO conducted the experiments. EJ, RJ, JM, and MG analyzed the data. All authors read and approved the manuscript.

## Conflict of Interest Statement

The authors declare that the research was conducted in the absence of any commercial or financial relationships that could be construed as a potential conflict of interest.

## Footnotes

1 https://www.ncdc.noaa.gov

2 http://co2now.org

3 https://blast.ncbi.nlm.nih.gov/Blast.cgi

4 https://www.ncbi.nlm.nih.gov/geo/query/acc.cgi?acc=GSE124940

## Appendix

Equations used to determine photosynthetic measurements.

P_n_: net photosynthesis rate (μmol/m^2^/s) for an open system

P_n_ = -W × C_o_ - C_i_ = -2005.39×((V ×P)/(T_a_×A))×(C_o_-C_i_)

Where:

C(C_i_): outlet (inlet) CO_2_ concentration (ppm or μmol/m^2^/s)

*W*: mass flow in mol/m^2^/s

*P*: atmospheric pressure (bar)

*T*_a_: air temperature (°C)

C_leaf_: leaf stomatal conductance (mmol/m^2^/s)

e_leaf_: saturated water vapor at leaf temperature (bar)

C_leaf_ = (W/(((e_leaf_-e_o_)/(e_o_-e_i_))×((P-e_o_)/(P-R_b_W))))×1000

e_leaf_ = 6.13753 × 10^-3^ × e^∧^(T_leaf_ × ((18.564 - (T_leaf_/254.4)/(T_leaf_ + 255.57))

Where:

*T_leaf_*: leaf temperature (°C)

*R_b_*: leaf boundary layer resistance (m^2^s/mol)

E: transpiration rate (mmol/m^2^/s)

E = ((e_o_ - e_i_)/(P - e_o_)) × W × 10^3^

e_o_ = ((hr_o_ × es)/100)

e_i_ = ((hr_i_ × es)/100)

e_s_ = 6.13753 × 10^-3^ ×e^∧^(Ta× (18.564 - ((Ta/254.4)/(Ta + 255.57)))

Where:

e_o_e_i_: outlet (inlet) water vapor (bar)

P: atmospheric pressure (bar)

e_s_: saturated water vapor at air temperature (bar)

T_a_: air temperature (°C)

hr(hr_i_): outlet (inlet) relative humidity (%)

T_leaf_: leaf temperature (°C)

R_b_: leaf boundary layer resistance (m^2^s/mol) – 0.3 m^2^s/mol is used

## Abbreviations

DPA: days post anthesis
GA: gibberellic acid
NILs: near isogenic lines
*Rht*: Reduced Height
RuBisCO: ribulose-1,5-bisphosphate carboxylase/oxygenase
SNP: single nucleotide polymorphism.
